# Frailty predicts need for medical review but not degree of organ support after complex orthopaedic surgery

**DOI:** 10.1186/cc13239

**Published:** 2014-03-17

**Authors:** N Singatoullina, A Panickar, A Dennis, R Porter, D Bryden

**Affiliations:** 1 Sheffield Teaching Hospitals, Sheffield, UK; 2 Univeristy of Leicester Hospitals, Leicester, UK

## Introduction

Based on expert opinion and case note review, the UK National Confidential Enquiry into Peri-operative Outcome has recommended provision of perioperative level 2 and 3 care to support major surgery in older people, and particularly those with comorbidity [1]. We wished to identify whether we could predict if the need was uniform and whether any factors could predict the degree of organ supports needed.

## Methods

A retrospective note review of all patients admitted to a level 2 critical care unit in the 12-month period from 1 January 2012 to 31 December 2012 undergoing revision hip surgery either as a two-stage or single-stage process. Surgery was undertaken at a national referral unit and chosen to represent an appropriate group of older, comorbid patients. Predefined preoperative and perioperative data were collected from chart review, along with postoperative physiological data whilst the patient was in critical care. This included frailty, comorbidities, operative blood loss, anaesthetic technique and level and duration or organ supports including the need for additional medical review whilst on the unit. Frailty was assessed preoperativelyusing the Rockwood assessment tool by trained staff [2]. Data were analysed using Microsoft Excel for Mac 2011 and Stata/IC 11.2 for Mac.

**Figure 1:**
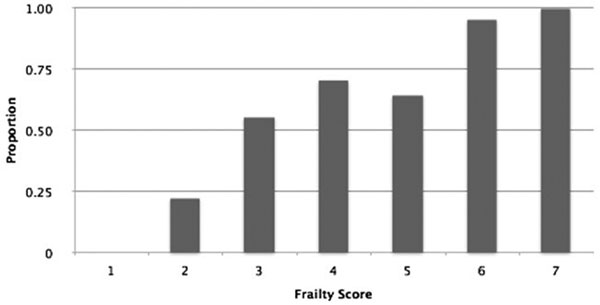
**Proportion of patients requiring additional medical review on critical care by degree of frailty**.

## Results

A total of 182 patients with a mean age of 69.8 years (range 29 to 92) were identified. Frail patients were significantly more likely to need additional medical input in the postoperative period whilst on critical care (Figure 1, *P *= 0.002) but this was not significantly linked to need for vasopressors, evidence of sepsis or choice of anaesthetic technique.

## Conclusion

In complex revision orthopaedic surgery, the need for postoperative level 2/3 support cannot be predicted from any preoperative or intraoperative factors but patient frailty does indicate the need for medical input in the postoperative period.

## References

[B1] Wilkinson K An Age Old Problem 2010 London: NCEPOD

[B2] Rockwood K CMAJ 2005 173 489 495 10.1503/cmaj.050051 16129869 PMC1188185

